# 2-(4-Chloro­phen­yl)-3-ethyl­sulfinyl-5-iodo-7-methyl-1-benzofuran

**DOI:** 10.1107/S1600536810051020

**Published:** 2010-12-11

**Authors:** Hong Dae Choi, Pil Ja Seo, Byeng Wha Son, Uk Lee

**Affiliations:** a Department of Chemistry, Dongeui University, San 24 Kaya-dong Busanjin-gu, Busan 614-714, Republic of Korea; b Department of Chemistry, Pukyong National University, 599-1 Daeyeon 3-dong Nam-gu, Busan 608-737, Republic of Korea

## Abstract

In the title compound, C_17_H_14_ClIO_2_S, the 4-chloro­phenyl ring makes a dihedral angle of 12.13 (5)° with the mean plane of the benzofuran ring. In the crystal, pairs of inter­molecular I⋯O contacts [3.145 (1) Å] link the mol­ecules into inversion dimers.

## Related literature

For the pharmacological activity of benzofuran compounds, see: Aslam *et al.* (2006 ▶); Galal *et al.* (2009 ▶); Khan *et al.* (2005 ▶). For natural products with benzofuran rings, see: Akgul & Anil (2003 ▶); Soekamto *et al.* (2003 ▶). For our previous structural studies of related 2-(4-chloro­phen­yl)-3-ethyl­sulfinyl-5-halo-1-benzofuran derivatives, see: Choi *et al.* (2010**a* ▶,b* ▶). For a review of halogen bonding, see: Politzer *et al.* (2007 ▶).
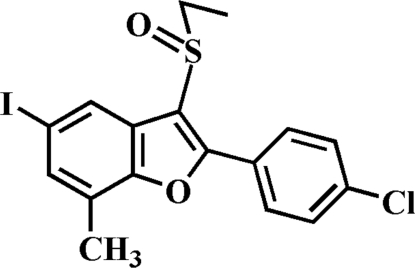


## Experimental

### 

#### Crystal data

C_17_H_14_ClIO_2_S
*M* *_r_* = 444.69
Triclinic, 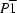

*a* = 7.3535 (1) Å
*b* = 10.4958 (2) Å
*c* = 11.9856 (2) Å
α = 68.509 (1)°
β = 88.317 (1)°
γ = 70.726 (1)°
*V* = 808.07 (2) Å^3^
*Z* = 2
Mo *K*α radiation
μ = 2.28 mm^−1^
*T* = 173 K
0.26 × 0.16 × 0.13 mm

#### Data collection

Bruker SMART APEXII CCD diffractometer
Absorption correction: multi-scan (*SADABS*; Bruker, 2009 ▶) *T*_min_ = 0.583, *T*_max_ = 0.746
14205 measured reflections
3738 independent reflections
3594 reflections with *I* > 2σ(*I*)
*R*_int_ = 0.024

#### Refinement

*R*[*F* ^2^ > 2σ(*F* ^2^)] = 0.020
*wR*(*F* ^2^) = 0.051
*S* = 1.09
3738 reflections
201 parameters
H-atom parameters constrained
Δρ_max_ = 0.45 e Å^−3^
Δρ_min_ = −0.94 e Å^−3^

### 

Data collection: *APEX2* (Bruker, 2009 ▶); cell refinement: *SAINT* (Bruker, 2009 ▶); data reduction: *SAINT*; program(s) used to solve structure: *SHELXS97* (Sheldrick, 2008 ▶); program(s) used to refine structure: *SHELXL97* (Sheldrick, 2008 ▶); molecular graphics: *ORTEP-3* (Farrugia, 1997 ▶) and *DIAMOND* (Brandenburg, 1998 ▶); software used to prepare material for publication: *SHELXL97*.

## Supplementary Material

Crystal structure: contains datablocks global, I. DOI: 10.1107/S1600536810051020/xu5113sup1.cif

Structure factors: contains datablocks I. DOI: 10.1107/S1600536810051020/xu5113Isup2.hkl

Additional supplementary materials:  crystallographic information; 3D view; checkCIF report

## Figures and Tables

**Fig. 1.**
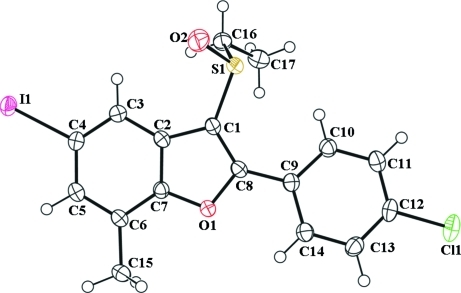
The molecular structure of the title compound with the atom numbering scheme. Displacement ellipsoids are drawn at the 50% probability level. H atoms are presented as a small spheres of arbitrary radius.

## supplementary crystallographic information

### Comment

Many compounds involving a benzofuran ring have attracted much attention owing to their potent pharmacological properties such as antifungal, antitumor and antiviral, antimicrobial activities (Aslam *et al.*, 2006, Galal *et al.*, 2009, Khan *et al.*, 2005). These compounds widely occur in nature (Akgul & Anil, 2003; Soekamto *et al.*, 2003). As a part of our continuing studies of the substituent effect on the solid state structures of 2-(4-chlorophenyl)-3-ethylsulfinyl-5-halo-1-benzofuran analogues (Choi *et al.*, 2010**a*,b*), we report herein on the crystal structure of the title compound.

In the title molecule (Fig. 1), the benzofuran unit is essentially planar, with a mean deviation of 0.020 (2) Å from the least-squares plane defined by the nine constituent atoms. The dihedral angle formed by the mean plane of the benzofuran ring and the 4-chlorophenyl ring is 12.13 (5)°. The molecular packing is stabilized by an I···O halogen-bonding between the iodine and the oxygen of the S═O unit [I1···O2^i^ = 3.145 (1) Å; C4—I1···O2^i^ = 169.00 (6)°; symmetry code; - *x*+1, - *y* +1, - *z* +1] (Politzer  *et al.*, 2007).

### Experimental

77% 3-Chloroperoxybenzoic acid (202 mg, 0.9 mmol) was added in small portions to a stirred solution of 2-(4-chlorophenyl)-3-ethylsulfanyl-5-iodo-7-methyl-1-benzofuran (343 mg, 0.8 mmol) in dichloromethane (30 mL) at 273 K. After being stirred at room temperature for 4 h, the mixture was washed with saturated sodium bicarbonate solution and the organic layer was separated, dried over magnesium sulfate, filtered and concentrated at reduced pressure. The residue was purified by column chromatography (hexane–ethyl acetate, 2:1 v/v) to afford the title compound as a colorless solid [yield 78%, m.p. 459–460 K;  *R*_f_ = 0.66 (hexane–ethyl acetate, 2:1 v/v)]. Single crystals suitable for X-ray diffraction were prepared by slow evaporation of a solution of the title compound in tetrahydofuran at room temperature.

### Refinement

All H atoms were positioned geometrically and refined using a riding model, with C—H = 0.95 Å for aryl, 0.99 Å  for methylene, and 0.98 Å for methyl H atoms. *U*_iso_(H) = 1.2*U*_eq_(C) for aryl and methylene H atoms, and 1.5*U*_eq_(C) for methyl H atoms.

### Crystal data

**Table:**

C_17_H_14_ClIO_2_S	*Z* = 2
*M**_r_* = 444.69	*F*(000) = 436
Triclinic, *P*1	*D*_x_ = 1.828 Mg m^−^^3^
Hall symbol: -P 1	Mo *K*α radiation, λ = 0.71073 Å
*a* = 7.3535 (1) Å	Cell parameters from 6925 reflections
*b* = 10.4958 (2) Å	θ = 2.6–27.4°
*c* = 11.9856 (2) Å	µ = 2.28 mm^−^^1^
α = 68.509 (1)°	*T* = 173 K
β = 88.317 (1)°	Block, colourless
γ = 70.726 (1)°	0.26 × 0.16 × 0.13 mm
*V* = 808.07 (2) Å^3^	

### Data collection

**Table:**

Bruker SMART APEXII CCD diffractometer	3738 independent reflections
Radiation source: rotating anode	3594 reflections with *I* > 2σ(*I*)
graphite multilayer	*R*_int_ = 0.024
Detector resolution: 10.0 pixels mm^-1^	θ_max_ = 27.6°, θ_min_ = 1.8°
φ and ω scans	*h* = −9→9
Absorption correction: multi-scan (*SADABS*; Bruker, 2009)	*k* = −13→13
*T*_min_ = 0.583, *T*_max_ = 0.746	*l* = −15→15
14205 measured reflections	

### Refinement

**Table:**

Refinement on *F*^2^	Primary atom site location: structure-invariant direct methods
Least-squares matrix: full	Secondary atom site location: difference Fourier map
*R*[*F*^2^ > 2σ(*F*^2^)] = 0.020	Hydrogen site location: difference Fourier map
*wR*(*F*^2^) = 0.051	H-atom parameters constrained
*S* = 1.09	*w* = 1/[σ^2^(*F*_o_^2^) + (0.0253*P*)^2^ + 0.4245*P*] where *P* = (*F*_o_^2^ + 2*F*_c_^2^)/3
3738 reflections	(Δ/σ)_max_ = 0.001
201 parameters	Δρ_max_ = 0.45 e Å^−^^3^
0 restraints	Δρ_min_ = −0.94 e Å^−^^3^

### Special details

**Table:**

Geometry. All esds (except the esd in the dihedral angle between two l.s. planes) are estimated using the full covariance matrix. The cell esds are taken into account individually in the estimation of esds in distances, angles and torsion angles; correlations between esds in cell parameters are only used when they are defined by crystal symmetry. An approximate (isotropic) treatment of cell esds is used for estimating esds involving l.s. planes.
Refinement. Refinement of F^2^ against ALL reflections. The weighted R-factor wR and goodness of fit S are based on F^2^, conventional R-factors R are based on F, with F set to zero for negative F^2^. The threshold expression of F^2^ > 2sigma(F^2^) is used only for calculating R-factors(gt) etc. and is not relevant to the choice of reflections for refinement. R-factors based on F^2^ are statistically about twice as large as those based on F, and R- factors based on ALL data will be even larger.

### Fractional atomic coordinates and isotropic or equivalent isotropic displacement parameters (Å^2^)

**Table:**

	*x*	*y*	*z*	*U*_iso_*/*U*_eq_	
I1	0.503893 (18)	0.274401 (13)	0.581925 (10)	0.02859 (5)	
Cl1	−0.07192 (8)	0.84278 (7)	−0.52118 (4)	0.03868 (12)	
S1	0.14553 (6)	0.80350 (5)	0.08547 (4)	0.02301 (9)	
O1	0.30263 (18)	0.42620 (14)	0.04254 (11)	0.0222 (2)	
O2	0.3030 (2)	0.80681 (17)	0.15921 (14)	0.0345 (3)	
C1	0.2076 (2)	0.62148 (19)	0.09517 (15)	0.0208 (3)	
C2	0.2943 (2)	0.49373 (19)	0.20294 (16)	0.0210 (3)	
C3	0.3318 (3)	0.4664 (2)	0.32484 (16)	0.0232 (3)	
H3	0.2922	0.5425	0.3548	0.028*	
C4	0.4289 (3)	0.3238 (2)	0.39943 (16)	0.0235 (3)	
C5	0.4907 (3)	0.2096 (2)	0.35746 (17)	0.0243 (3)	
H5	0.5592	0.1135	0.4125	0.029*	
C6	0.4537 (3)	0.2344 (2)	0.23693 (16)	0.0226 (3)	
C7	0.3527 (2)	0.3779 (2)	0.16434 (15)	0.0209 (3)	
C8	0.2147 (2)	0.57512 (19)	0.00175 (16)	0.0208 (3)	
C9	0.1473 (3)	0.6442 (2)	−0.12649 (16)	0.0215 (3)	
C10	0.0170 (3)	0.7870 (2)	−0.17795 (17)	0.0254 (4)	
H10	−0.0272	0.8422	−0.1289	0.030*	
C11	−0.0488 (3)	0.8489 (2)	−0.29937 (17)	0.0279 (4)	
H11	−0.1361	0.9464	−0.3339	0.033*	
C12	0.0141 (3)	0.7673 (2)	−0.36959 (16)	0.0266 (4)	
C13	0.1431 (3)	0.6251 (2)	−0.32175 (17)	0.0282 (4)	
H13	0.1848	0.5703	−0.3714	0.034*	
C14	0.2101 (3)	0.5646 (2)	−0.20065 (17)	0.0256 (4)	
H14	0.2999	0.4678	−0.1673	0.031*	
C15	0.5208 (3)	0.1159 (2)	0.18763 (18)	0.0287 (4)	
H15A	0.4093	0.0931	0.1687	0.043*	
H15B	0.6139	0.0285	0.2478	0.043*	
H15C	0.5829	0.1488	0.1142	0.043*	
C16	−0.0614 (3)	0.8112 (2)	0.17132 (18)	0.0297 (4)	
H16A	−0.0306	0.7209	0.2445	0.036*	
H16B	−0.0915	0.8948	0.1971	0.036*	
C17	−0.2369 (3)	0.8279 (2)	0.0956 (2)	0.0325 (4)	
H17A	−0.2731	0.9210	0.0264	0.049*	
H17B	−0.3455	0.8260	0.1448	0.049*	
H17C	−0.2048	0.7476	0.0669	0.049*	

### Atomic displacement parameters (Å^2^)

**Table:**

	*U*^11^	*U*^22^	*U*^33^	*U*^12^	*U*^13^	*U*^23^
I1	0.03639 (8)	0.02645 (8)	0.01946 (7)	−0.01066 (6)	−0.00220 (5)	−0.00457 (5)
Cl1	0.0389 (3)	0.0503 (3)	0.0189 (2)	−0.0157 (2)	−0.00118 (19)	−0.0037 (2)
S1	0.0265 (2)	0.0185 (2)	0.0227 (2)	−0.00738 (17)	0.00088 (17)	−0.00659 (17)
O1	0.0241 (6)	0.0192 (6)	0.0197 (6)	−0.0044 (5)	0.0011 (5)	−0.0062 (5)
O2	0.0367 (8)	0.0346 (8)	0.0356 (8)	−0.0170 (6)	−0.0046 (6)	−0.0121 (6)
C1	0.0201 (8)	0.0186 (8)	0.0202 (8)	−0.0049 (6)	0.0012 (6)	−0.0050 (7)
C2	0.0187 (8)	0.0189 (8)	0.0229 (8)	−0.0051 (6)	0.0014 (6)	−0.0063 (7)
C3	0.0236 (8)	0.0229 (9)	0.0232 (8)	−0.0076 (7)	0.0019 (7)	−0.0092 (7)
C4	0.0236 (8)	0.0259 (9)	0.0197 (8)	−0.0091 (7)	0.0008 (6)	−0.0064 (7)
C5	0.0230 (8)	0.0202 (9)	0.0244 (8)	−0.0056 (7)	0.0004 (7)	−0.0040 (7)
C6	0.0203 (8)	0.0194 (8)	0.0248 (8)	−0.0046 (7)	0.0019 (7)	−0.0066 (7)
C7	0.0193 (8)	0.0217 (9)	0.0199 (8)	−0.0059 (7)	0.0017 (6)	−0.0068 (7)
C8	0.0189 (8)	0.0180 (8)	0.0227 (8)	−0.0049 (6)	0.0031 (6)	−0.0060 (7)
C9	0.0208 (8)	0.0221 (9)	0.0210 (8)	−0.0094 (7)	0.0026 (6)	−0.0057 (7)
C10	0.0239 (9)	0.0256 (9)	0.0236 (9)	−0.0059 (7)	0.0021 (7)	−0.0083 (7)
C11	0.0238 (9)	0.0258 (10)	0.0260 (9)	−0.0056 (7)	0.0010 (7)	−0.0034 (8)
C12	0.0264 (9)	0.0338 (10)	0.0173 (8)	−0.0148 (8)	0.0011 (7)	−0.0033 (7)
C13	0.0337 (10)	0.0311 (10)	0.0222 (9)	−0.0139 (8)	0.0060 (7)	−0.0106 (8)
C14	0.0286 (9)	0.0230 (9)	0.0233 (9)	−0.0094 (7)	0.0033 (7)	−0.0063 (7)
C15	0.0310 (10)	0.0214 (9)	0.0293 (9)	−0.0033 (7)	0.0026 (8)	−0.0096 (8)
C16	0.0308 (10)	0.0283 (10)	0.0262 (9)	−0.0038 (8)	0.0058 (8)	−0.0120 (8)
C17	0.0282 (10)	0.0314 (11)	0.0404 (11)	−0.0111 (8)	0.0090 (8)	−0.0158 (9)

### Geometric parameters (Å, °)

**Table:**

I1—C4	2.0995 (17)	C9—C10	1.397 (3)
I1—O2^i^	3.1451 (14)	C9—C14	1.403 (3)
Cl1—C12	1.7351 (18)	C10—C11	1.384 (3)
S1—O2	1.4940 (14)	C10—H10	0.9500
S1—C1	1.7709 (18)	C11—C12	1.378 (3)
S1—C16	1.808 (2)	C11—H11	0.9500
O1—C7	1.375 (2)	C12—C13	1.388 (3)
O1—C8	1.375 (2)	C13—C14	1.383 (3)
C1—C8	1.368 (2)	C13—H13	0.9500
C1—C2	1.445 (2)	C14—H14	0.9500
C2—C7	1.390 (2)	C15—H15A	0.9800
C2—C3	1.399 (2)	C15—H15B	0.9800
C3—C4	1.379 (3)	C15—H15C	0.9800
C3—H3	0.9500	C16—C17	1.524 (3)
C4—C5	1.402 (3)	C16—H16A	0.9900
C5—C6	1.389 (3)	C16—H16B	0.9900
C5—H5	0.9500	C17—H17A	0.9800
C6—C7	1.385 (3)	C17—H17B	0.9800
C6—C15	1.502 (2)	C17—H17C	0.9800
C8—C9	1.458 (2)		
			
C4—I1—O2^i^	169.00 (6)	C11—C10—H10	119.5
O2—S1—C1	106.72 (9)	C9—C10—H10	119.5
O2—S1—C16	107.26 (9)	C12—C11—C10	119.13 (18)
C1—S1—C16	98.39 (9)	C12—C11—H11	120.4
C7—O1—C8	106.98 (13)	C10—C11—H11	120.4
C8—C1—C2	107.22 (15)	C11—C12—C13	121.62 (17)
C8—C1—S1	127.20 (14)	C11—C12—Cl1	119.41 (16)
C2—C1—S1	124.72 (13)	C13—C12—Cl1	118.97 (15)
C7—C2—C3	119.17 (16)	C14—C13—C12	118.90 (18)
C7—C2—C1	105.01 (15)	C14—C13—H13	120.5
C3—C2—C1	135.80 (17)	C12—C13—H13	120.5
C4—C3—C2	116.85 (16)	C13—C14—C9	120.86 (18)
C4—C3—H3	121.6	C13—C14—H14	119.6
C2—C3—H3	121.6	C9—C14—H14	119.6
C3—C4—C5	122.78 (16)	C6—C15—H15A	109.5
C3—C4—I1	119.43 (13)	C6—C15—H15B	109.5
C5—C4—I1	117.69 (13)	H15A—C15—H15B	109.5
C6—C5—C4	121.23 (17)	C6—C15—H15C	109.5
C6—C5—H5	119.4	H15A—C15—H15C	109.5
C4—C5—H5	119.4	H15B—C15—H15C	109.5
C7—C6—C5	114.88 (16)	C17—C16—S1	110.43 (13)
C7—C6—C15	122.11 (16)	C17—C16—H16A	109.6
C5—C6—C15	122.99 (17)	S1—C16—H16A	109.6
O1—C7—C6	124.33 (16)	C17—C16—H16B	109.6
O1—C7—C2	110.58 (15)	S1—C16—H16B	109.6
C6—C7—C2	125.05 (16)	H16A—C16—H16B	108.1
C1—C8—O1	110.19 (15)	C16—C17—H17A	109.5
C1—C8—C9	135.59 (16)	C16—C17—H17B	109.5
O1—C8—C9	114.19 (15)	H17A—C17—H17B	109.5
C10—C9—C14	118.53 (16)	C16—C17—H17C	109.5
C10—C9—C8	122.01 (16)	H17A—C17—H17C	109.5
C14—C9—C8	119.44 (16)	H17B—C17—H17C	109.5
C11—C10—C9	120.95 (17)		
			
O2—S1—C1—C8	−129.18 (16)	C3—C2—C7—C6	−2.4 (3)
C16—S1—C1—C8	119.88 (17)	C1—C2—C7—C6	176.07 (17)
O2—S1—C1—C2	38.84 (17)	C2—C1—C8—O1	−0.8 (2)
C16—S1—C1—C2	−72.10 (16)	S1—C1—C8—O1	168.86 (12)
C8—C1—C2—C7	1.57 (19)	C2—C1—C8—C9	176.90 (19)
S1—C1—C2—C7	−168.46 (13)	S1—C1—C8—C9	−13.4 (3)
C8—C1—C2—C3	179.7 (2)	C7—O1—C8—C1	−0.23 (18)
S1—C1—C2—C3	9.7 (3)	C7—O1—C8—C9	−178.50 (14)
C7—C2—C3—C4	1.0 (2)	C1—C8—C9—C10	−12.8 (3)
C1—C2—C3—C4	−176.88 (19)	O1—C8—C9—C10	164.87 (16)
C2—C3—C4—C5	0.6 (3)	C1—C8—C9—C14	169.0 (2)
C2—C3—C4—I1	176.89 (13)	O1—C8—C9—C14	−13.3 (2)
C3—C4—C5—C6	−1.1 (3)	C14—C9—C10—C11	−0.1 (3)
I1—C4—C5—C6	−177.44 (13)	C8—C9—C10—C11	−178.32 (17)
C4—C5—C6—C7	−0.1 (3)	C9—C10—C11—C12	0.8 (3)
C4—C5—C6—C15	178.68 (18)	C10—C11—C12—C13	−0.7 (3)
C8—O1—C7—C6	−176.56 (17)	C10—C11—C12—Cl1	178.54 (15)
C8—O1—C7—C2	1.28 (18)	C11—C12—C13—C14	−0.2 (3)
C5—C6—C7—O1	179.43 (16)	Cl1—C12—C13—C14	−179.37 (15)
C15—C6—C7—O1	0.6 (3)	C12—C13—C14—C9	0.9 (3)
C5—C6—C7—C2	1.9 (3)	C10—C9—C14—C13	−0.7 (3)
C15—C6—C7—C2	−176.90 (17)	C8—C9—C14—C13	177.52 (17)
C3—C2—C7—O1	179.75 (15)	O2—S1—C16—C17	172.76 (14)
C1—C2—C7—O1	−1.76 (19)	C1—S1—C16—C17	−76.74 (16)

Symmetry codes: (i) −*x*+1, −*y*+1, −*z*+1.
